# Does the *GNB3* C825T Polymorphism Influence Swimming Performance in Competitive Swimmers?

**DOI:** 10.1515/hukin-2015-0075

**Published:** 2015-10-14

**Authors:** Agata Grenda, Marek Sawczuk, Mariusz Kaczmarczyk, Agnieszka Maciejewska, Danuta Umiastowska, Wioletta Łubkowska, Piotr Żmijewski, Paweł Cięszczyk

**Affiliations:** 1 Department of Physical Education and Sport, West Pomeranian Technological University, Szczecin, Poland.; 2 University of Szczecin, Department of Physical Culture and Health Promotion, Szczecin, Poland.; 3 Pomeranian Medical University, Department of Clinical and Molecular Biochemistry, Szczecin, Poland.; 4 Institute of Sport, Warszawa, Poland.; 5 Academy of Physical Education and Sport, Department of Sport Education, Gdansk, Poland.

**Keywords:** GNB3, gene polymorphism, swimming

## Abstract

Single nucleotide polymorphism C825T located within the GNB3 gene has been proposed in the literature as the performance enhancing polymorphism in highly trained athletes. Therefore, the aim of the present study was to verify the hypothesis assuming an association between the C825T polymorphic site and performance of competitive swimmers. The frequencies of C/T alleles and distribution of CC, CT and TT genotypes of the C825T GNB3 polymorphism were compared between athletes and nonathletic controls as well as between sprint and endurance swimmers. Genomic DNA was extracted from 197 competitive swimmers (50 long distance swimmers (LDS) and 147 short distance swimmers (SDS)) and 379 sedentary volunteers. The allele frequencies and genotype distribution of the C825T polymorphic site were not significantly different when LDS and SDS were compared to sedentary controls. Gender-specific analysis did not reveal any significant differences in allele and genotype distribution, neither between female controls and female swimmers nor between male controls and male swimmers. No significant differences in allele frequencies and genotype distribution were observed when LDS and SDS as well as groups of swimmers stratified by gender were compared. The results of this study do not support the hypothesis that the C825T polymorphism of the GNB3 gene is associated with swimming performance in competitive swimmers.

## Introduction

Concurrence of various exo- and endogenous factors is required to achieve the highest athletic performance level (Macarthur and North, 2005). The main exogenous factors are specialized training, nutrition and proper supplementation. The endogenous factors include all components of the human body which are engaged in physical activity e.g. coordination, physique, thermoregulation, exercise metabolism, water and electrolyte balance as well as psychological factors (Łubkowska and Troszczynski, 2014). All of these components are, to a greater or lesser degree, determined by genetic factors that significantly influence the inter-subject differences in adapting to the applied training and achievement of the champion status by only a few athletes. That is why it is thought that allelic versions of certain genes in the athlete’s genotype (preferred in a given sports discipline) are one of the elements enabling achievement of the highest possible level of performance. One of the genes that has been proposed as a potential candidate for physical performance is the GNB3 (Eynon et al., 2009). It encodes the subunit beta 3 of guanine nucleotide binding protein that belongs to the heterotrimeric G-proteins family. These complexes are connected with metabotropic receptors and are responsible for the transduction of cell signals between receptors and intracellular effectors (Cabrera-Vera et al., 2003). The β 3 subunit is expressed in all human tissues and is one of the key elements of the intracellular signal transmission system. Within the GNB3 that is located in chromosome 12, many polymorphisms have been reported (Klenke and Siffert, 2011). The functional C (cytosine) to T (thymine) single nucleotide polymophism (SNP) referred to as C825T (rs5443) deserves special attention taking into consideration its influence on the function of the coded polypeptide. This C→T transition in position 825 of the cDNA of the gene is associated with the creation of an alternative splicing site within exon 9 of the GNB3 and leads to the removal of 41 amino acids from the target polypeptide sequence of the β3 subunit. It was established that a shorter isoform of the beta 3 subunit, encoded by the 825T allele, is associated with enhanced activity of the entire G-protein complex and results in increased transduction of intracellular signals (Siffert et al., 1995, 1998). The conducted research points to the relation between the C825T polymorphism of the GNB3 gene with the pathophysiology of many disorders of the circulatory system as hypertension or left ventricular hypertrophy (Schunkert et al., 1998; Poch et al., 2000), emphasizing the role of the allele T and the genotype TT as risk factors for such diseases (Siffert et al., 1998). Other analyses suggest that the described polymorphic site may have influence on the volume of adipose tissue, heart rate, and blood pressure in response to endurance training (Rankinen et al., 2002), as well as the ability to absorb oxygen in untrained individuals (Faruque et al., 2009). Research conducted on a group of top level Israeli athletes revealed a higher frequency of the genotype TT in elite endurance athletes in comparison to controls and athletes practicing strength-speed sports (Eynon et al., 2009). However, the results of these observations have not been confirmed in subsequent research by Ruiz et al. (2011) and Sawczuk et al. (2014).

Taking into consideration the ambiguity of previous genetic association research results, we decided to verify the hypothesis assuming an association between this polymorphic site and the phenotype of competitive swimmers. For this purpose, we conducted analyses aimed at establishing the frequency of genotypes and alleles in competitive swimmers divided into two groups (long distance swimmers - LDS, and short distance swimmers - SDS), as well as compared the resulting frequencies between individual athlete groups and a non-training control group.

## Material and Methods

### Ethics Committee

The Pomeranian Medical University Ethics Committee, Poland, approved the study and an informed consent form was completed by each participant. The study complied with the guidelines set out in the Declaration of Helsinki and the ethics policy of the Szczecin University (Kruk, 2013).

### Participants

One hundred and ninety-seven Polish competitive swimmers (104 males and 93 females, 20.35 ± 2.58 years), who competed in national and international events, were recruited for this study. All participants were Caucasians to minimize the influence of racial genetic skew and to avoid any potential population stratification problems. The swimmers were divided into two groups, based on their competitive distance and values of relative contribution of the aerobic or/anaerobic energy systems: long distance swimmers (LDS, n=50, 24 males, 26 females), more than 500 m (aerobic events) and short distance swimmers (SDS, n=147, 80 males, 67 females), between 50 and 200 m (predominantly anaerobic events).

All investigated swimmers had been finalists of the Polish National Championships. Additionally, 7 of them had participated in the Olympic Games and 48 of them had taken part in the World Championships or European Championships. A control group of healthy individuals (n = 379, 222 males and 157 females, 22.6 ± 2.8 years) was also selected from the Polish population (college students) with no background in swimming.

### Genetic analyses

The buccal cells donated by the participants were collected in Resuspension Solution (GenElute Mammalian Genomic DNA Miniprep Kit, Sigma, Germany) using sterile foam-tipped applicators (Puritan, USA). DNA was extracted from the buccal cells using a GenElute Mammalian Genomic DNA Miniprep Kit according to the manufacturer’s protocol.

All samples were genotyped using an allelic discrimination assay on a CFX96 Touch™ Real-Time PCR Detection System (Biorad, USA) with TaqMan probes. For discrimination between the *GNB3* C825 and 825T (rs rs5443) alleles, TaqMan Pre-Designed SNP Genotyping Assays were used (Applied Biosystems, USA), including primers and fluorescently labelled (FAM and VIC) MGB probes.

### Statistical analysis

The STATISTICA statistical package (version 8.0, StatSoft Inc., Tulsa, Oklahoma, USA) was used to perform all analyses. The Hardy-Weinberg equilibrium (HWE) for *GNB3* (rs5443) genotypes was assessed separately in swimmers and control subjects with the Pearson’s chi-squared test. Genotype distributions as well as allele frequencies were determined and chi-square tests were used to compare the *GNB3* C825 alleles and genotypes frequencies between the groups of athletes and control participants. The analysis of the individual effects of these variants was based on three genetic models: general, dominant and recessive. The odds ratio (OR) with 95% confidence intervals was calculated. Bonferroni’s correction for multiple testing was applied, and the alpha level for determining statistical significance was set at p < 0.016.

## Results

The *GNB3* C825T genotype distributions of all athletes including the LDS group and SDS group as well as sedentary controls met Hardy-Weinberg equilibrium (all p > 0.025). The results of the distribution of alleles and genotypes for the *GNB3* C825T polymorphic site in competitive athletes compared with the non-training controls (all subjects stratified by gender) are presented in Table 1. The genotype of the C825T polymorphic site was not significantly different when long distance swimmers (n=50, 52.0% CC, 40.0% CT, 8.0% TT) and short distance swimmers (n=147, 43.5% CC, 45.6% CT, 10.9% TT) were compared to sedentary controls (n=379, 44.6% CC, 46.7% CT, 8.7% TT; p= 0.562 and p=0.743, respectively). The frequencies of C and T alleles in the control group (C/T=67.9/32.1) were also similar to these of the LDS (C/T=72.0/28.0) and SDS group (C/T=66.3/33.7) and did not reach statistical significance (p=0.480 and p=0.668, respectively).

Gender-specific analysis did not reveal any significant differences in genotype distribution, neither between female controls (n=157, 43.3% CC, 47.8% CT, 8.9% TT) and female swimmers (n=93, 47.3% CC, 43.0% CT, 9.7% TT, p=0.766) nor between male controls (n=222, 45.5% CC, 45.9% CT, 8.6% TT) and male swimmers (n=104, 44.2% CC, 45.2% CT, 10.6% TT, p=0.841). Similarly, no significant differences in allele frequencies were noted among male (p=0.0.742)/ female (p=0.783) swimmers and respective control groups (Table 1). The data showed genotype-dependent differences in the likelihood of being classified as a long distance swimmer under general, dominant and recessive models, both in male and female LDS. Specifically, long distance swimmers were less likely to posses the recessive T allele or TT genotype (all ORs calculated below 1.0). However, the p values were not statistically significant (Table 2).

When all long distance swimmers and all short distance swimmers were compared, no significant differences in allele frequencies and genotype distribution were observed (p=0.355 and p = 0.562, respectively). Neither significant differences were noticed in alleles and genotypes distribution when groups of swimmers stratified by gender were compared (male LDS vs male SDS, p=0.619, p=0.791; female LDS vs female SDS, p=0.545, p=0.724)

## Discussion

The current research represents a genetic case-control study, approach that is widely applied in identifying genotype:phenotype associations. Therefore, to test the hypothesis assuming an association between the C825T polymorphic site of the GNB3 and the phenotype of competitive swimmers, we compared genotype distribution and frequencies of alleles among swimmers (divided into two groups: long distance swimmers and short distance swimmers) and non-athletic controls being a random representation of the general population. The main findings of the study are as follows: i) during the research, we found the analyzed group of athletes and the control group in genetic balance, ii) the research did not show any statistically significant differences in the frequency of both the alleles and genotypes of the C825T polymorphism of the GNB3 gene between the studied groups and controls. The acquired data did not indicate any association between the frequency of alleles and genotypes of the C825T polymorphism and the sex of the subjects. Thus, we did not corroborate the findings of Eynon et al. (2009) that the GNB3 TT genotype was more frequent in endurance-oriented athletes than in sprinters. However, we confirmed the observations of Ruiz et al. (2011) and Sawczuk et al. (2014) in which no correlation between the frequency of alleles and genotypes of the C825T polymorphism and athletic status was observed.

Since our research did not yield any statistically significant higher frequency of either of the analyzed alleles (C and T) or genotypes (CC, CT, and TT) in the studied swimmers, it seems necessary to reject the hypothesis assuming an association between the C825T polymorphism of the GNB3 gene and the phenotype of swimmers. It may be suspected that the presence of individual C825T allelic variants of the GNB3 gene does not increase or decrease the probability that its carriers will develop physiological or metabolic features which might be relevant for highly qualified athletes such as competitive swimmers.

This effect may be modulated or compensated, individually or in the haplotype, by other allelic variants within the area of the GNB3 gene. Since genes and their products are never functionally isolated and they influence one another in a complex net of interdependencies, it also cannot be ruled out that other genetic loci influence the functions of the beta 3 subunit of guanine nucleotide binding proteins that belong to the heterotrimeric G-proteins family and have an impact on differences in the biochemical-physiological properties of the body. In fact, there have been attempts to test the hypothesis of the potential interaction between the C825T polymorphism of the GNB3 and other polymorphic variants (9bp long −9/+9, rs5810761) of the bradykinin receptor b2 gene (BDKRB2), that were previously associated with elite athletic performance (Eynon et al., 2011a). However, the authors, did not observe any association of these two genes in relation to endurance performance. Recent research has demonstrated that the phenotype of an elite athlete is highly polygenic; so far, attempts have been made to assess the relation between at least 80 genetic markers and the properties of the body which influence athletic performance (Ruiz et al., 2009, 2010; Ahmetov and Fedotovskaya, 2012). For instance, it has been estimated that it is almost impossible for endurance athletes to posses the optimal DNA variants for endurance performance and it would be expected that only few individuals exist with 16 favorable allelic variants of all 23 “endurance” polymorphisms, but probably no individuals with 17 genotypes in the world population (Williams and Folland, 2008). Swimming performance is a complex trait that depends on numerous phenotypes, not only muscle contractility, blood oxygen transport capacity, cardiopulmonary function as well as fatty acids and glucose metabolism. It seems that many gene loci, each with a small contribution, rather than single gene polymorphic variants with substantial effects on individual variation of human athletic performance are responsible for swimming performance (Grenda et al., 2014a, 2014b). Thus, it is inevitable for sport genetics to focus on analysis of complex interaction of multiple genetic polymorphisms that enhance sport related phenotypes as well as alterations to the genome that do not involve changes in the nucleotide sequences of the genes. The last one called epigenetics includes all “external” chromatin modifications. It is thought that the role of DNA methylation and histone acetylation in gene availability for transcriptional machinery as well as miRNA as post-transcriptional regulators of the protein synthesis in determination of inter-individual variability in response to applied physical exercises is probably equally important as changes in the DNA sequence (Eynon et al., 2011b; Ruiz et al., 2011; Voisin et al., 2015). The present study has certain limitations that hamper the progression of other genetic case-control association studies of athletic performance i.e. small groups of athletes of the highest possible level in their given discipline. Therefore, multisite collaborations and data-sharing between researchers will be necessary, to ensure sufficient statistical power of athletic-based genetic research (Eynon et al., 2014; Sawczuk et al., 2015).

## Conclusion

In conclusion, we did not provide the evidence for an association between the GNB3 C825T polymorphism and swimming performance in a group of competitive swimmers as we did not obtain any statistically significantly higher frequency of either of the analyzed alleles (C and T) or genotypes (CC, CT and TT) in the studied group of athletes.

It may be suspected that harboring of C825T allelic variants of the GNB3 gene neither decreases nor increases the probability of being a competitive swimmer, and the GNB3 gene cannot be considered a factor allowing understanding individual variations in exercise-related phenotypes.

## Practical implications

The analyses performed in the present research are aimed primarily at increasing sports science knowledge by explanations of new facts and phenomena. Regardless of being not statistically significant, our results should facilitate understanding the impact of genetic component on sport related phenotypes. We hope that based on knowledge about specific DNA markers as well as epigenetic modifications analyzed in genetic studies, it will be possible to create genetic tests which enable the identification of sport talents or the personalization and maximization of training quality in order to achieve optimal results.

## Figures and Tables

**Table 1:** Frequencies of the GNB3 gene C825T genotypes and alleles in swimmers and controls. p values are calculated by χ^2^ test and χ^2^ test with Yates' correction for comparisons between groups of athletes and the control group for genotypes and alleles, respectively

	*GNB3* C825T genotypes						MAF		

Group	CC (%)	CT (%)	TT (%)	p	p	HWE	(% of allele T	p	p
**Male swimmers** (n=104)	46 (44.2)	47 (45.2)	11 (10.6)	0.841†		0.844	33.2	0.742†	
*Long distance swimmers (n=24)*	12 (50.0)	10 (41.7)	2 (8.3)	0.863†	0.791‡	0.967	29.2	0.862†	0.619‡
*Short distance swimmers (n=80)*	34 (42.6)	37 (46.2)	9 (11.2)	0.970†	1.000	0.822	34.4	0.575†	1.000
**Male controls (n=222)**	101 (45.5)	102 (45.9)	19 (8.6)	1.000		0.340	31.5	1.000	
**Female swimmers (n=93)**	44 (47.3)	40 (43.0)	9 (9.7)	0.766†		0.983	31.2	0.783†	
*Long distance swimmers (n=26)*	14 (53.8)	10 (38.5)	2 (7.7)	0.604†	0.724‡	0.908	26.9	0.495†	0.545‡
*Short distance swimmers (n=67)*	30 (44.8)	30 (44.8)	7 (10.4)	0.891†	1.000	0.901	32.8	0.916†	1.000
**Female controls (n=157)**	68 (43.3)	75 (47.8)	14 (8.9)	1.000		0.295	32.8	1.000	
**All swimmers (n=197)**	90 (45.7)	87 (44.2)	20 (10.1)	0.774†		0.879	32.2	0.998†	
*Long distance swimmers (n=50)*	26 (52.0)	20 (40.0)	4 (8.0)	0.609†	0.562‡	0.955	28.0	0.480†	0.355‡
*Short distance swimmers (n=147)*	64 (43.5)	67 (45.6)	16 (10.9)	0.743†	1.000	0.805	33.7	0.668†	1.000
**All controls (n=379)**	169 (44.6)	177 (46.7)	33 (8.7)	1.000		0.160	32.1	1.000	

MAF – minor allele frequency. HWE – exact test

† versus Controls

‡ versus Short distance swimmers

**Table 2:** Odds ratios (OR), confidential intervals (CI) and p values for codominant, dominant and recessive genetic models in each swimmer group stratified by gender

Genetic model										
	**Control**	***LDS***	OR	p	***SDS***	OR	p	***LDS + SDS***	OR	p
	**Male**									
*Codominant*										
CC	101	12	1.00	1.000	34	1.00	1.000	46	1.00	1.000
CT	102	10	0.82	0.670	37	1.08	0.787	47	1.01	0.963
TT	19	2	0.87	0.880	9	1.41	0.448	11	1.27	0.566
*Dominant*										
CC	101	12	1.00	1.000	34	1.00	1.000	46	1.00	1.000
CT+TT	121	12	0.83	0.674	46	1.13	0.644	58	1.05	0.831
*Recessive*										
CC+CT	203	22		1.000	71	1.00	1.000	93	1.00	1.000
TT	19	2	0.97	0.970	9	1.35	0.478	11	1.26	0.557
	**Female**									
*Codominant*										
CC	68	14	1.00	1.000	30	1.00	1.000	44	1.00	1.000
CT	75	10	0.65	0.331	30	0.91	0.750	40	0.82	0.482
TT	14	2	0.69	0.652	7	1.13	0.807	9	0.99	0.989
*Dominant*										
CC	68	14	1.00	1.000	30	1.00	1.000	44	1.00	1.000
CT+TT	89	12	0.65	0.319	37	0.94	0.840	49	0.85	0.539
*Recessive*										
CC+CT	143	24	1.00	1.000	60	1.00	1.000	84	1.00	1.000
TT	14	2	0.85	0.838	7	1.19	0.719	9	1.09	0.841
	**Male+Female**									
*Codominant*										
CC	169	26	1.00	1.000	64	1.00	1.000	90	1.00	1.000
CT	177	20	0.73	0.329	67	0.99	0.998	87	0.92	0.665
TT	33	4	0.79	0.676	16	1.28	0.465	20	1.13	0.678
*Dominant*										
CC	169	26	1.00	1.000	64	1.00	1.000	90	1.00	1.000
CT+TT	210	24	0.74	0.324	83	1.04	0.827	107	0.96	0.802
*Recessive*										
CC+CT	346	46	1.00	1.000	131	1.00	1.000	177	1.00	1.000
TT	33	4	0.91	0.867	16	1.28	0.442	20	1.18	0.570
